# Telephone Administration of the Human Activity Profile Questionnaire in Patients With Cardiovascular Disease: Methodological Study

**DOI:** 10.2196/75164

**Published:** 2025-12-31

**Authors:** Tamara Costabile Sant´Anna, Júlia Isaac Bernardes, Adriana Marques Alcici Moreira, Janaine Cunha Polese, Maria Da Glória Rodrigues-Machado

**Affiliations:** 1 Faculdade de Ciências Médicas de Minas Gerais, Alameda Ezequiel Dias, 275 - Centro, Belo Horizonte/MG, 30130-110, Brazil, +55 3134987275; 2 Universidade Federal de Minas Gerais, Belo Horizonte, Brazil

**Keywords:** physical functional performance, cardiovascular rehabilitation, surveys and questionnaires, telephone call, interview

## Abstract

**Background:**

The Human Activity Profile (HAP) questionnaire is widely used to assess functional capacity in patients with chronic diseases. However, its remote administration via telephone has not been validated in individuals with cardiovascular disease (CVD), despite the increasing need for accessible assessment methods, particularly in contexts involving mobility limitations or reduced access to in-person care.

**Objective:**

We aimed to validate administration of the HAP questionnaire via telephone in patients with CVD who were participating in a cardiovascular rehabilitation program.

**Methods:**

This methodological study included 56 patients with CVD (36/56, 64% men; mean age 75.14, SD 10.28 years). Participants completed the HAP twice, once face-to-face and once by telephone, with a 3- to 14-day interval. Maximum activity score (MAS) and adjusted activity score (AAS) were analyzed. Internal consistency was assessed using Cronbach α, test-retest reliability was assessed using intraclass correlation coefficients (ICCs), and agreement between modalities was assessed using Bland-Altman plots. Paired comparisons between modes were performed using mean differences (MDs) and *P* values.

**Results:**

MAS values did not differ significantly between face-to-face and telephone administration (mean 79.11, SD 11.48 vs mean 82.71, SD 7.48; MD –3.61; *P*=.10). AAS values showed a similar pattern (mean 69.11, SD 14.18 vs mean 71.21, SD 13.43; MD –2.11; *P*=.05). Internal consistency was excellent (Cronbach α=0.919), and reliability was high (ICC=0.794 for MAS; ICC=0.910 for AAS). Bland-Altman analyses demonstrated acceptable limits of agreement for MAS (–19.3 to 12.1) and AAS (–17.6 to 13.4), with no systematic bias.

**Conclusions:**

The HAP questionnaire administered by telephone demonstrates high reliability, excellent internal consistency, and strong agreement with face-to-face application. Telephone-based administration represents a valid, practical, and accessible alternative for assessing functional capacity in patients with cardiovascular disease, particularly when in-person evaluations are not feasible.

## Introduction

Despite a sustained decline in cardiovascular disease (CVD) morbidity and mortality over the past 4 decades, CVD remains a leading cause of disease burden worldwide [1-3]. Nearly 80% of CVDs occur in low- and middle-income countries, where aging and urbanization trends have accelerated the increase in the burden of CVD [4].

Functional capacity assessment is an important part of clinical evaluation, especially in patients with chronic conditions such as heart failure, lung disease, neuromuscular disease, and other conditions that may affect functionality [3,5-8]. Maximal exercise tests are widely used in functional capacity assessment, but the high cost may limit their use. Thus, a possible alternative is the application of scales and questionnaires that constitute relatively simple and economical tools, do not require sophisticated equipment or specialized environments to be administered, and are more accessible to a greater number of patients [8].

The Human Activity Profile (HAP) is a simple questionnaire designed to assess functional capacity in relation to daily activities and physical fitness and can offer a more accessible, convenient, and cost-effective approach to assessment [9]. The HAP questionnaire quantifies functional capacity individually, providing relevant information about physical well-being and supporting strategies to promote active lifestyles and monitor disease progression [10,11].

The HAP questionnaire has demonstrated validity in cardiovascular populations and has been used alongside reference measures such as cardiopulmonary exercise testing that is considered the gold standard for assessing functional capacity, as well as with the 6-Minute Walk Test, which is widely applied in clinical settings, showing good correlation with functional capacity [10,11]. In populations with Chagas cardiomyopathy and heart failure, the HAP questionnaire has been associated with measures such as functional class and systolic function, reinforcing its clinical relevance in characterizing functional limitations in CVD [12]. Additionally, its application has proven reliable across diverse clinical settings, including hospitalized and community-dwelling older adults [9,13,14].

In recent years, the application of questionnaires by telephone has gained prominence as a viable method of data collection, especially during periods requiring social distancing, such as the COVID-19 pandemic. Studies have successfully validated the use of telephone-based tools, including functional assessments such as the Duke Activity Status Index, in individuals with CVD and stroke [15-17]. Such methods facilitate rapid and remote evaluations, reducing barriers for patients with mobility limitations or restricted access to in-person care.

Therefore, applying the HAP questionnaire via telephone may represent a practical alternative for remote assessment of functional capacity in individuals with CVD.

The objective of this study is to validate the telephone administration of the HAP questionnaire in patients with CVD who were participating in a cardiovascular rehabilitation program.

## Methods

### Study Design

This is a methodological study designed to assess the reliability and validity of the HAP questionnaire when administered via telephone to patients with CVD. The study followed the Consensus-Based Standards for the Selection of Health Measurement Instruments guidelines [18], which recommend a minimum of 50 participants for studies assessing measurement properties such as reliability and construct validity. This sample size is considered adequate to detect moderate to high intraclass correlation coefficients (ICCs; ICC >0.70) with sufficient statistical power in validation studies.

### Ethical Considerations

The study was approved by the institutional research ethics committee (number 5.456.711) and was conducted in accordance with the ethical standards outlined in the Declaration of Helsinki. All participants provided written informed consent before inclusion in the study.

### Participants

Participants were recruited from a cardiovascular rehabilitation program, and the sample was composed of patients diagnosed with CVD. A convenience sampling method was used on the basis of availability and eligibility of patients enrolled in the program during the study period.

The inclusion criteria were individuals of both sexes who were aged between 50 and 90 years and with a confirmed diagnosis of CVD. The exclusion criteria included patients with auditory or cognitive impairments that could interfere with communication during the telephone interview and those who failed to complete the second part of the questionnaire within the maximum interval of 14 days between assessments.

A total of 56 participants met the eligibility criteria and completed both the face-to-face and telephone assessments. Demographic, clinical, and pharmacological characteristics of the sample are presented in Table 1.

**Table 1.** Sample characterization (N=56).

Variables	Sample
Sex (male), n (%)	36 (64)
Age (years), mean (SD)	75.14 (10.28)
Weight (kg), mean (SD)	73.02 (13.64)
Height (cm), mean (SD)	165.29 (10.11)
BMI (kg/m²), mean (SD)	26.65 (3.97)
Medication use, n (%)	
Antihypertensives	48 (86)
Statins	44 (79)
Beta blockers	26 (47)
Anticoagulants	22 (39)
Antiglycemic agents	12 (22)
Cardiovascular diagnosis, n (%)	
Coronary artery disease	31 (55)
Arrhythmia	15 (27)
Acute myocardial infarction	7 (13)
Valvulopathy	3 (5)
Congestive heart failure	2 (4)
Comorbidities, n (%)	
Dyslipidemia	55 (98)
Systemic arterial hypertension	54 (96)
Obesity	13 (23)
Diabetes mellitus	11 (19)
Chronic kidney disease	2 (4)
Stroke	1 (2)
Procedure or surgery performed, n (%)	
Stent placement	15 (27)
Coronary artery bypass grafting	1 (2)

### HAP Questionnaire

The HAP questionnaire is composed of 94 items that assess an individual’s level of physical function across domains such as self-care, work, social engagement, and physical activity. The items are ordered by increasing metabolic demand, with higher item numbers representing activities that require greater energy expenditure [9,11].

Participants were asked to classify each activity as one they are “still doing,” have “stopped doing,” or have “never done.” The HAP questionnaire can be applied to individuals with a wide range of functional capacities—from very low (eg, getting in and out of bed without assistance) to very high (eg, running 4.8 kilometers) [9,11].

The questionnaire generates 2 primary scores:

- Maximum activity score (MAS): the number of the most strenuous activity the participant is still performing
- Adjusted activity score (AAS): calculated by subtracting the number of activities the participant has “stopped doing” from the MAS, up to and including the activity with the highest score still performed

The AAS is considered a more stable estimate of the individual’s typical functional level compared to the MAS, as it adjusts for discontinued activities. According to established cutoffs, AAS values categorize individuals as: inactive (AAS<53), moderately active (AAS=53‐74), and active (AAS>74) [9,14].

### Procedure

The HAP questionnaire was administered to each participant on 2 separate occasions: once in person and once via telephone. The interval between the two assessments ranged from 3 to 14 days (mean 8, SD 4.5 d), which was deemed appropriate to minimize recall bias while avoiding significant changes in clinical status [19].

Each participant completed both modes of administration only once, and both applications were conducted by the same trained physiotherapist who followed a standardized protocol to ensure consistency in tone, pacing, and instructions across both modalities.

The order of administration was not randomized, with all participants first completing the face-to-face assessment followed by the telephone interview. This fixed order was chosen to maximize adherence and reduce the risk of dropout, particularly among older participants, based on preliminary feasibility observations during pilot testing.

### Statistical Analysis

Categorical variables were reported as absolute frequencies and percentages, whereas continuous variables were expressed as means and SDs. To evaluate convergent validity, the mean differences (MDs) between face-to-face and telephone assessments were calculated along with 95% CIs.

Cronbach α was used to assess internal consistency, with values greater than 0.70 considered acceptable. The ICC was applied to determine test-retest reliability and temporal stability of the HAP questionnaire across administration modes. The magnitude of ICC was classified as follows: very high (>0.90), high (0.70‐0.89), moderate (0.50‐0.69), low (0.26‐0.49), and very low (<0.25) [19].

To assess agreement between the two methods, the Bland-Altman method was used, comparing the mean values and the differences between in-person and telephone assessments for MAS and AAS. The upper and lower limits of agreement were calculated as MD −1.96 to +1.96 times the SD of the differences, and plotted to visualize the dispersion and potential bias.

All statistical analyses were performed using R software (version 1.0), and a significance level of 5% (*P*<.05) was adopted.

## Results

**Figure 1.**
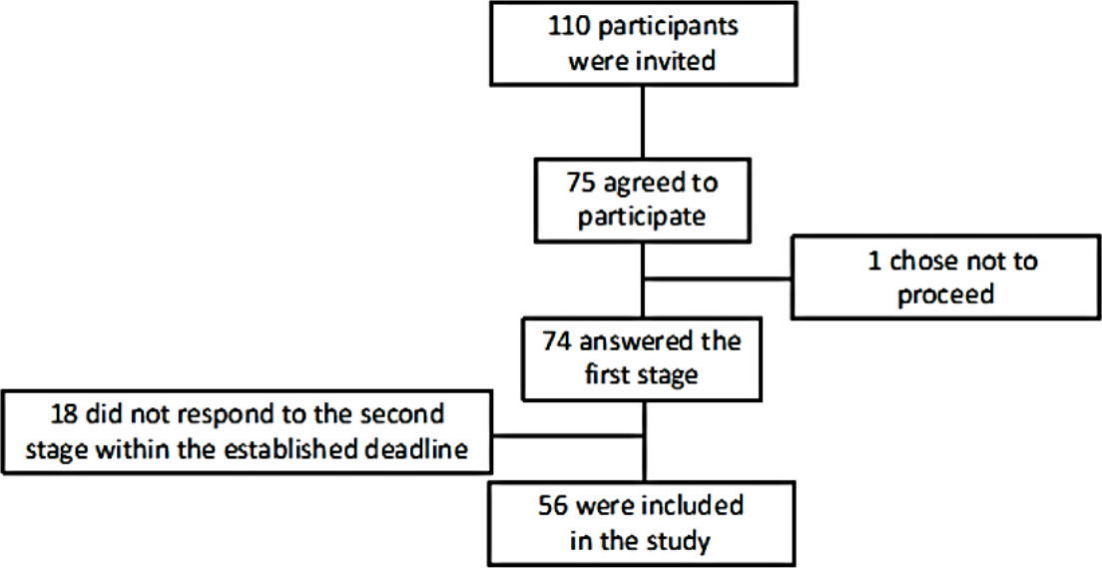
Flowchart of participant recruitment and inclusion in the study. The figure illustrates the recruitment process, including the number of eligible individuals, those who consented to participate, those who completed the first assessment, and those who were ultimately included in the final sample after completing both face-to-face and telephone evaluations. Reasons for exclusion—such as dropout or failure to complete the second assessment within 14 days—are also indicated.

Participants were recruited between October 10, 2022, and July 31, 2023. A total of 110 individuals were eligible, of whom 75 signed the informed consent form, and 1 participant withdrew before data collection. In total, 74 individuals completed the first phase of the study. However, 18 participants did not complete both assessments—either failing to respond to the phone call or to attend the face-to-face assessment within the required 14-day interval. Thus, 56 participants completed both modes of questionnaire administration and were included in the final analysis (Figure 1).

The final sample included both men and women, with a mean age of 75.14 (SD 10.8) years. The most prevalent cardiovascular risk factors were dyslipidemia (55/56, 98.2%) and systemic arterial hypertension (54/56, 96.4%), as shown in Table 1. These characteristics reflect a clinically representative population for validation of functional assessment tools in individuals with CVD [18].

Internal consistency and temporal stability of the HAP were assessed using Cronbach α and ICC, respectively. The results demonstrated excellent internal consistency for both MAS and AAS, with Cronbach α=0.919 (95% CI 0.879‐0.948). Test-retest reliability was classified as high for MAS (ICC=0.794; 95% CI 0.649‐0.879) and very high for AAS (ICC=0.910; 95% CI 0.847‐0.947), as shown in Table 2.

There were no statistically significant differences between face-to-face and telephone applications for either MAS (*P*=.10) or AAS (*P*=.05), as shown in Table 3. This supports the equivalence of the 2 administration methods.

**Table 2.** Internal consistency and test-retest reliability of the Human Activity Profile questionnaire.

Score	ICC^a^	95% CI for ICC	Cronbach α	95% CI for Cronbach α
MAS^b^	0.794	0.649‐0.879	0.919	0.879‐0.948
AAS^c^	0.910	0.847‐0.947	—^d^	—

a ICC: intraclass correlation coefficient.

b MAS: maximum activity score.

c AAS: adjusted activity score.

d Not applicable.

**Table 3.** Comparison of the Human Activity Profile questionnaire scores between face-to-face and telephone administration.

Score	Face-to-face, mean (SD)	Telephone, mean (SD)	Mean difference	*P* value
MAS^a^	79.11 (11.48)	82.71 (7.48)	–3.61	.10
AAS^b^	69.11 (14.18)	71.21 (13.43)	–2.11	.05

a MAS: maximum activity score.

b AAS: adjusted activity score.

**Figure 2.**
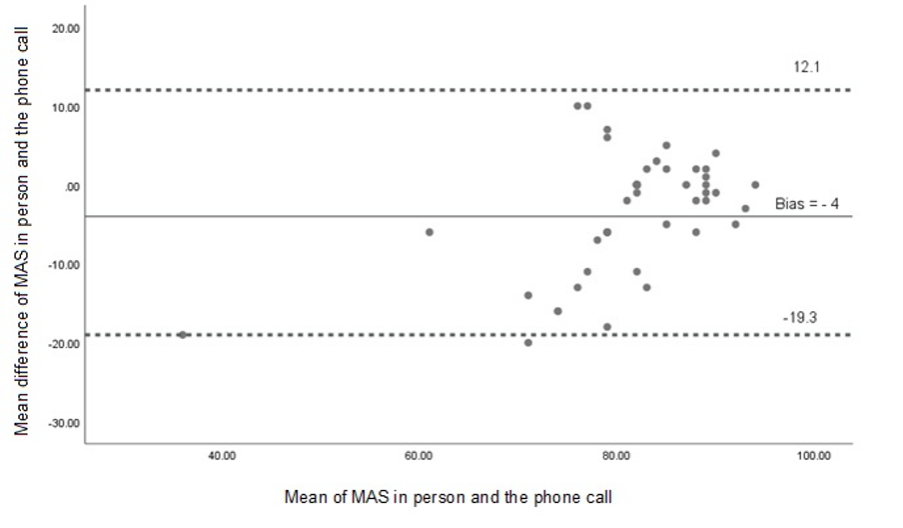
Bland-Altman plot showing agreement between maximum activity scores (MASs) obtained through face-to-face and telephone administration of the Human Activity Profile questionnaire. The plot illustrates the mean difference between MASs obtained from face-to-face and telephone assessments, along with the 95% limits of agreement (mean difference –1.96 to +1.96 SDs), represented by dotted lines. No systematic bias was observed, and most data points lie within the limits of agreement, indicating acceptable agreement between modes.

Agreement between modalities was further examined using Bland-Altman analysis. For MAS, the mean bias was –4.0 (95% CI –19.3 to 12.1) and –2.1 for AAS (95% CI –17.6 to 13.4), indicating acceptable agreement without systematic error, as shown in Figures2  3. Linear regression analysis showed nonsignificant slopes for both MAS and AAS (*P*>.05), confirming the absence of proportional bias.

**Figure 3.**
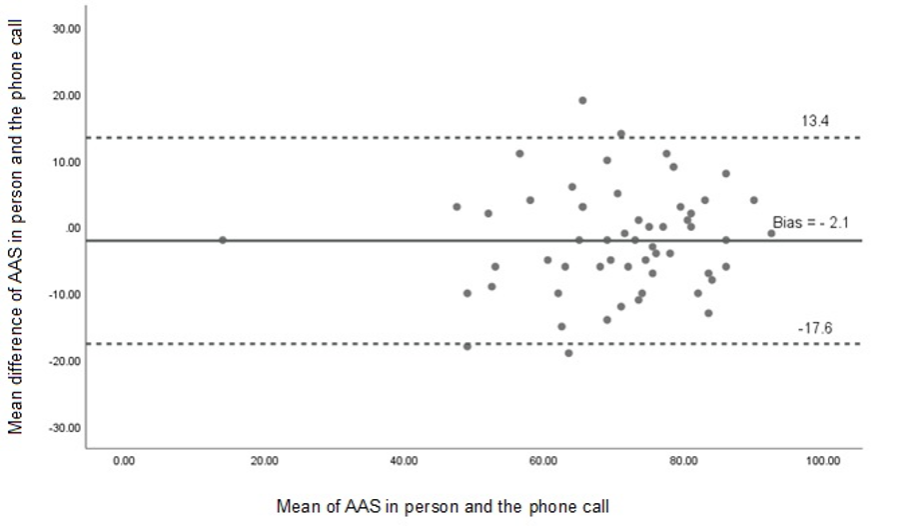
Bland-Altman plot showing agreement between adjusted activity scores (AASs) obtained through face-to-face and telephone administration of the Human Activity Profile questionnaire. The plot displays the mean difference between AASs obtained via face-to-face and telephone assessments, along with the 95% limits of agreement (mean difference –1.96 to +1.96 SDs), indicated by dotted lines. The distribution of data points within the limits suggests acceptable agreement and no evidence of systematic bias between the 2 modes of administration.

Table 4 presents the distribution of participants across functional capacity categories (inactive, moderately active, and active) based on AASs. Results were consistent across both assessment modes, reinforcing the reproducibility of classification.

No participants scored the minimum or maximum possible values for MASs (0 or 94) or AASs (1 or 94). Therefore, no floor or ceiling effects were observed, indicating that the HAP questionnaire demonstrated adequate responsiveness in this sample.

**Table 4.** Classification of physical activity level by adjusted activity score (AAS) in face-to-face and telephone questionnaire administration (N=56).

Category	Face-to-face		Telephone	
	Frequency, n (%)	Mean AAS (SD)	Frequency, n (%)	Mean AAS (SD)
Inactive	7 (12.5)	42.42 (6.33)	4 (7.1)	40.75 (10.05)
Moderately active	24 (42.9)	64.68 (5.89)	24 (42.9)	64.87 6.42)
Active	25 (44.6)	80.33 (4.72)	28 (50.0)	81.00 (5.22)

a MAS: maximum activity score.

## Discussion

### Principal Findings

This study demonstrated that the HAP questionnaire administered by telephone yields results highly consistent with those obtained through face-to-face application in patients with CVD. Both MASs and AASs showed no notable differences between modalities, and the instrument exhibited excellent internal consistency (Cronbach α=0.919), strong test-retest reliability (ICC=0.794 for MAS; ICC=0.910 for AAS), and acceptable agreement on Bland-Altman analysis with no systematic bias. These findings indicate that telephone administration is a valid, reliable, and practical alternative for assessing functional capacity in this population, supporting the feasibility of remote functional evaluation [10,11,13,14].

Building upon these findings, the HAP questionnaire offers additional advantages that reinforce its applicability in clinical and research settings. The questionnaire allows for quick and meaningful measurements of activity level changes, comparisons with healthy populations, and identification of activity patterns associated with medical conditions [11]. It has shown correlations with objective measures of cardiorespiratory fitness [20] and has been validated for use in various populations, such as individuals with chronic pain and knee osteoarthritis [21].

In this study, the questionnaire demonstrated good internal consistency, with a Cronbach α of 0.919, and strong reproducibility and temporal stability measured by the ICC, with MAS ICC=0.794 and AAS ICC=0.910. These findings align with previous validation studies. For example, a systematic review reported high consistency for MAS (0.76‐0.97) and AAS (0.79‐0.97), reinforcing the questionnaire’s measurement reliability [13].

The Cronbach α of 0.919 in this study is considered excellent and indicative of near-perfect reliability [22]. Similar values have been observed in previous research; one study reported a Cronbach α of 0.91 when applying the HAP questionnaire via interview in a community-dwelling older adult population [9], and another study found a Cronbach α of 0.93 in hospitalized patients [14], reinforcing the questionnaire’s internal consistency across diverse populations.

The internal consistency and temporal stability did not substantially differ between face-to-face and telephone administrations, suggesting that both methods are equally reliable. Our in-person results are consistent with previous studies; one investigation reported MAS and AAS of 71.6 and 63.6, respectively, in patients undergoing coronary artery bypass grafting [11], whereas another study observed an MAS of 79.76 and an AAS of 65.81 in patients with heart failure [10]. Importantly, this study is, to our knowledge, the first to evaluate both the internal consistency and the agreement of HAP questionnaire scores when administered in person versus by telephone.

The Bland-Altman analysis confirmed good agreement between administration methods. For MAS, the limits of agreement (95% CI) ranged from −19.3 to 12.1, and from −17.6 to 13.4 for AAS. These findings suggest that the differences between the modes are within acceptable limits, with no significant measurement bias introduced by the telephone modality. No previous studies have reported such agreement data for the HAP questionnaire using this method of comparison.

Several previous studies have successfully used telephone-based instruments in patients with CVD [15,16] and in poststroke populations [17,23]. For instance, one study assessed pharmacist-led telephone-based education and self-management support during the COVID-19 pandemic lockdown [15,23]. Another investigation examined angina prevalence among stable outpatients with CVD using remote methods [16], and a separate validation study confirmed the reliability of the Duke Activity Status Index administration by telephone in stroke survivors [17]. Telephone administration allows for faster, more direct data collection and extends access to individuals who may be unable to attend face-to-face assessments. To our knowledge, this is the first study to validate the use of the HAP questionnaire via telephone in patients with CVD.

### Strength and Limitations of the Study

The assessment of physical activity levels is a key component of both in-person and home-based exercise programs. Validating a tool like the HAP questionnaire for telephone administration broadens access to functional capacity assessment, particularly for individuals living in rural or remote areas, those with limited mobility, or patients who are unable to attend in-person sessions. This approach also facilitates multicenter research by enabling efficient and cost-effective data collection across geographically diverse populations. Additionally, traditional functional tests often require specialized equipment or trained professionals, representing a high financial burden and logistical barrier, particularly for underserved populations [24-27].

However, this study has some limitations. Certain patients may face challenges related to hearing loss, language comprehension, or cognitive deficits, which can interfere with telephone-based assessment. Moreover, the lower technological literacy commonly observed among older adults, combined with socioeconomic and ethnic disparities, may further limit the widespread applicability of telephone-based tools [28].

### Conclusions

The results of this study demonstrated that the HAP questionnaire, when administered by telephone, shows adequate reliability, agreement, and internal consistency, supporting its use as a valid alternative for assessing physical activity levels in patients with CVD. These findings suggest that telephone-based administration of the HAP questionnaire may be a practical, accessible, and cost-effective tool for functional assessment in clinical and research contexts, especially when in-person evaluations are not feasible.

## Abbreviations

AAS: adjusted activity score
CVD: cardiovascular disease
HAP: Human Activity Profile
ICC: interclass correlation coefficient
MAS: maximum activity score
MD: mean difference

## References

[1] GBD 2019 Diseases and Injuries Collaborators (2020). Global burden of 369 diseases and injuries in 204 countries and territories, 1990–2019: a systematic analysis for the Global Burden of Disease Study 2019. Lancet.

[2] Ruivo J, Moholdt T, Abreu A (2023). Overview of Cardiac Rehabilitation following post-acute myocardial infarction in European Society of Cardiology member countries. Eur J Prev Cardiol.

[3] Virani SS, Newby LK, Writing Committee Members (2023). 2023 AHA/ACC/ACCP/ASPC/NLA/PCNA Guideline for the Management of Patients With Chronic Coronary Disease: a report of the American Heart Association/American College of Cardiology Joint Committee on Clinical Practice Guidelines. J Am Coll Cardiol.

[4] Li Y, Cao GY, Jing WZ, Liu J, Liu M (2023). Global trends and regional differences in incidence and mortality of cardiovascular disease, 1990-2019: findings from 2019 global burden of disease study. Eur J Prev Cardiol.

[5] Anderson L, Sharp GA, Norton RJ (2017). Home-based versus centre-based cardiac rehabilitation. Cochrane Database Syst Rev.

[6] Rostagno C, Gensini GF (2008). Six minute walk test: a simple and useful test to evaluate functional capacity in patients with heart failure. Intern Emerg Med.

[7] Wijeysundera DN, Pearse RM, Shulman MA (2018). Assessment of functional capacity before major non-cardiac surgery: an international, prospective cohort study. Lancet.

[8] Ferguson M, Shulman M (2022). Cardiopulmonary exercise testing and other tests of functional capacity. Curr Anesthesiol Rep.

[9] Souza AC, Magalhães LC, Teixeira-Salmela LF (2006). Cross-cultural adaptation and analysis of the psychometric properties in the Brazilian version of the Human Activity Profile. Cad Saude Publica.

[10] Ribeiro-Samora GA, Pereira DA, Vieira OA (2016). Using the human activity profile to assess functional performance in heart failure. J Cardiopulm Rehabil Prev.

[11] Parry M, Arthur H, Brooks D, Groll D, Pavlov A (2012). Measuring function in older adults with co-morbid illnesses who are undergoing coronary artery bypass graft (CABG) surgery. Arch Gerontol Geriatr.

[12] Costa HS, Lima MM, Vieira CF (2021). Assessment of functional performance in Chagas heart disease by Human Activity Profile questionnaire. Disabil Rehabil.

[13] Davidson M, de Morton N (2007). A systematic review of the Human Activity Profile. Clin Rehabil.

[14] Souza DC, Wegner F, Costa LC, Chiavegato LD, Lunardi AC (2017). Measurement properties of the Human Activity Profile questionnaire in hospitalized patients. Braz J Phys Ther.

[15] Gona OJ, Madhan R, Shambu SK (2020). Assessment of clinical pharmacists’ assistance for patients with established cardiovascular diseases during the COVID-19 pandemic: insights from southern India. Front Cardiovasc Med.

[16] Blumenthal DM, Howard SE, Searl Como J (2021). Prevalence of angina among primary care patients with coronary artery disease. JAMA Netw Open.

[17] Dias C, Torriani-Pasin C, Galvão AC, Costa PH, Polese JC (2023). Validation of the Duke Activity Status Index questionnaire by telephone in individuals after stroke. Int J Cardiol Cardiovasc Risk Prev.

[18] Cohen J (1988). Set correlation and contingency tables. Appl Psychol Meas.

[19] Avelino PR, Nascimento LR, Menezes KK (2021). Validation of the telephone-based assessment of locomotion ability after stroke. Int J Rehabil Res.

[20] Daughton DM, Fix AJ, Kass I, Bell CW, Patil KD (1982). Maximum oxygen consumption and the ADAPT quality-of-life scale. Arch Phys Med Rehabil.

[21] Bennell KL, Hinman RS, Crossley KM (2004). Is the Human Activity Profile a useful measure in people with knee osteoarthritis?. J Rehabil Res Dev.

[22] Landis JR, Koch GG (1977). The measurement of observer agreement for categorical data. Biometrics.

[23] Bilek LD, Camp KL, Dawn DM, Dvorak M (2000). Evaluation of the Human Activities Profile Questionnaire for use with persons with arthritis. Arthritis Care Res.

[24] Canada JM, Reynolds MA, Myers R (2021). Usefulness of the Duke Activity Status Index to select an optimal cardiovascular exercise stress test protocol. Am J Cardiol.

[25] Pook M, Elhaj H, El Kefraoui C (2022). Construct validity and responsiveness of the Duke Activity Status Index (DASI) as a measure of recovery after colorectal surgery. Surg Endosc.

[26] Abbasi-Ghahramanloo A, Soltani-Kermanshahi M, Mansori K (2020). Comparison of SF-36 and WHOQoL-BREF in measuring quality of life in patients with type 2 diabetes. Int J Gen Med.

[27] Govil N, Parag K, Kumar B, Khandelwal H, Dua R, Sivaji P (2020). Translation, cultural adaptation, and validation of the Duke Activity Status Index in the Hindi language. Ann Card Anaesth.

[28] Atlas A, Muru-Lanning M, Moyes S, Kerse N, Jatrana S (2020). Cell phone and technology use by octogenarians. J Prim Health Care.

